# Changes in remnant cholesterol levels after coronary angiography: inflammation and cardiovascular risk assessment

**DOI:** 10.1590/1806-9282.20250423

**Published:** 2025-09-19

**Authors:** Reşat Dikme, Mahmut Padak, Yusuf Güler, Ayşegül Sarpkaya, Ömer Göç, Ömer Faruk Çiçek

**Affiliations:** 1 Harran University, Vocational School of Health Services, Dialysis Program – Şanlıurfa, Turkey.; 2 Harran University, Faculty of Health Sciences, Department of Perfusion – Şanlıurfa, Turkey.; 3 Mehmet Akif Inan Training and Research Hospital, Department of Perfusion – Şanlıurfa, Turkey.; 4 Harran University, Vocational School of Health Services, Podology Program – Şanlıurfa, Turkey.; 5 Harran University, Vocational School of Health Services, Biomedical Device Technology Program – Şanlıurfa, Turkey.; 6 Mehmet Akif Inan Training and Research Hospital, Department of Cardiology – Şanlıurfa, Turkey.

**Keywords:** Cholesterol, Angiography, Inflammation, CRP, Lipid metabolism, Troponin T

## Abstract

**OBJECTIVE:**

Remnant cholesterol is an important component of triglyceride-rich lipoproteins and has emerged as an important potential biomarker for the assessment of cardiovascular risk. The aim of the study is to examine acute changes in remnant cholesterol levels pre- and post-angiography and evaluate their relationship with inflammatory markers.

**METHODS:**

A total of 183 patients who underwent coronary angiography were included in this retrospective study. Remnant cholesterol, low-density lipoprotein, high-density lipoprotein, creatine kinase-myocardial band, Troponin-T, C-reactive protein, and white blood cell levels were analyzed pre- and post-angiography. Correlation and regression analyses were used to evaluate the relationships between the data.

**RESULTS:**

Remnant cholesterol levels increased significantly post-angiography (p<0.05), while low-density lipoprotein showed no significant change (p=0.0588). C-reactive protein levels were significantly higher post-angiography than pre-angiography (p<0.05), which was considered as an indicator of procedure-related inflammatory response. Correlation analysis found a significant inverse relationship between baseline remnant cholesterol and post-angiography C-reactive protein (p<0.05). However, in a multivariable regression, C-reactive protein did not have a significant independent effect on remnant cholesterol levels (p>0.05). Baseline remnant cholesterol (pre-angiography) levels showed an inverse association with post-procedure troponin T (β=-0.014, p<0.05), whereas post-angiography remnant cholesterol demonstrated no significant correlation with troponin T (p>0.05).

**CONCLUSION:**

This study reveals significant post-angiography increases in remnant cholesterol, indicating acute metabolic and inflammatory changes. While these findings enhance understanding of remnant cholesterol dynamics during vascular interventions, the lack of long-term outcomes necessitates cautious interpretation. Future research should validate its prognostic value and therapeutic implications. Remnant cholesterol shows potential as a biomarker but requires further investigation before clinical implementation.

## INTRODUCTION

Remnant cholesterol, derived from triglyceride-rich lipoproteins like chylomicrons and very low-density lipoproteins (VLDL), is a key contributor to atherosclerosis and cardiovascular disease^1^. Elevated levels increase the risk of myocardial infarction, ischemic stroke, and peripheral arterial disease, independent of low-density lipoprotein (LDL)^2,3^. Remnant cholesterol promotes plaque accumulation and arterial stenosis, with higher levels observed in affected individuals^4^. It also triggers inflammation, as evidenced by its correlation with biomarkers like C-reactive protein (CRP), accelerating atherosclerosis through inflammatory processes^5^.

Unlike LDL, remnant cholesterol can directly cross the arterial intima and be taken up by macrophages without oxidation, accumulating in arterial walls and promoting plaque formation. It is metabolized by endothelial lipase, releasing toxic metabolites that induce endothelial dysfunction and low-grade inflammation^6,7^.

Remnant cholesterol is a promising therapeutic target for preventing acute coronary syndrome and atherosclerotic heart disease. Lifestyle changes and medical therapies aimed at reducing remnant cholesterol levels may effectively lower cardiovascular risks^1,6^.

Coronary angiography, a key diagnostic and treatment tool for atherosclerotic cardiovascular diseases, was studied in patients with advanced atherosclerosis and significant inflammation. This study retrospectively analyzed pre- and post-coronary angiography remnant cholesterol levels in patients with advanced atherosclerosis, aiming to evaluate its role as a cardiovascular risk marker and optimize treatment strategies.

## METHODS

### Study design and patient selection

In this retrospective study, we analyzed the biochemical data of 183 patients over 18 years of age who underwent coronary angiography in Şanlıurfa Mehmet Akif İnan Training and Research Hospital between 2022 (January) and 2024 (September). Demographic data, biochemical total cholesterol, high-density lipoprotein (HDL), LDL, creatine kinase-MB (CK-MB), troponin T, CRP, and white blood cell (WBC) results were obtained retrospectively from hospital records. Ethics committee approval (date 18.11.2024, HRÜ/24.18.21) was obtained, and the study was conducted in accordance with the Declaration of Helsinki. According to the records, post-angiography laboratory measurements were performed within 18–24 h following the procedure, using morning fasting blood samples. In all patients, a minimum of 12 h of fasting was ensured for both pre- and post-angiography measurements, and samples were processed according to standard protocols. All peri-procedural medications, including interventions during the angiography and routinely administered heparin, were recorded. To minimize the effect of heparin on post-procedure lipid measurements, the blood sampling time was planned to be at least 12 h after heparin administration.

### Remnant cholesterol measurement

Remnant cholesterol levels in plasma are determined through two main methods: calculation and direct measurement^8^. The calculated method involves subtracting the sum of LDL and HDL from total cholesterol^5,7^, offering a practical, cost-effective, and widely applicable approach in clinical settings without requiring specialized equipment. Direct measurement methods, such as ultracentrifugation, nuclear magnetic resonance spectroscopy, and automated assays, use enzymes and surfactants to quantify total cholesterol in chylomicrons and VLDL^9,10^. In this study, the calculation method was chosen due to its simplicity, reliance on routine lipid panel results, rapid application, and alignment with international guidelines and clinical research^5,7^.

### Statistical analysis

Both parametric and nonparametric statistical methods were employed to analyze the study data. Normality was assessed using the Shapiro-Wilk test. For normally distributed data, the dependent sample t-test was applied, while the Wilcoxon signed-rank test was used for non-normally distributed data. Correlation analyses were conducted to examine relationships between variables: Pearson correlation for normally distributed data and Spearman correlation for non-normally distributed data. Additionally, multiple regression analysis was performed to assess causal relationships between dependent and independent variables. To examine potential associations, multiple linear regression models were constructed. These models do not imply causality but aim to describe the statistical relationships between variables at different time points. A statistical significance level of p<0.05 was adopted. All analyses were conducted using Statistical Package for the Social Sciences (SPSS) (v25.0) and Python (v3.X) software.

## RESULTS

A total of 183 patients (105 males, 78 females) were included in the study, with a mean age of 61.57±12.39 years. According to the available data, patients who underwent elective diagnostic coronary angiography and were diagnosed with stable angina pectoris were included in the study, while patients with acute coronary syndrome (ACS) were excluded. Upon reviewing the patients’ underlying comorbidities, it was found that 42 patients (22.9%) had diabetes, 34 patients (18.6%) had hypertension, and 21 patients (11.5%) had both diabetes and hypertension. During the angiography procedure, 132 patients (72%) received stent placements, while 24 patients (13%) underwent balloon angioplasty.

The levels of biochemical variables pre- and post-angiography and the statistical significance and test statistics of these changes are given in Table 1.

**Table 1:** Comparison of biochemical variables pre- and post-angiography.

Variable	Pre-angiography Mean±SD	Post-angiography Mean±SD	Test statistic	p-value
Remnant cholesterol (mg/dL)	32.14±17.37	43.96±24.23	3.27	**0.0017**
Total cholesterol (mg/dL)	190.02±44.06	196.13±33.79	710.5	0.1316
HDL (mg/dL)	39.30±9.12	39.97±6.10	777	0.2256
LDL (mg/dL)	118.57±41.47	112.19±41.18	682.5	0.0588
CK-MB (μg/L)	62.93±118.48	11.14±12.98	179	**3.62×10^-8^**
Troponin-T (ng/L)	253.63±241.91	362.43±485.06	730	0.1213
CRP (mg/dL)	6.20±5.11	11.22±13.13	443.5	**0.0003**
WBC (10³/μL)	9.70±2.43	9.50±2.33	852	0.5018

Normality was assessed using the Shapiro-Wilk test. A paired sample t-test was applied to normally distributed variables (remnant cholesterol, total cholesterol, HDL, LDL, and WBC), while the Wilcoxon signed-rank test was used for non-normally distributed variables (CK-MB, troponin-T, and CRP). SD: standard deviation; HDL: high-density lipoprotein; LDL: low-density lipoprotein; CK-MB: creatine kinase-MB; CRP: C-reactive protein; WBC: white blood cell. Bold values indicate statistical significance at p<0.05.

As seen in Table 1, remnant cholesterol and CRP levels significantly increased (p<0.05), while CK-MB levels were significantly lower post-angiography than pre-angiography (p<0.001). These changes highlight the effects of angiography on lipid metabolism and inflammation. However, no significant changes were observed in troponin-T and HDL levels. Although LDL levels showed a numerical decrease post-angiography, the change was not statistically significant (p=0.0588).

Correlation analysis was performed to evaluate the relationships between the data in the study. Table 2 summarizes the statistically significant and clinically important correlations.

**Table 2:** Significant correlations between biochemical parameters pre- and post-angiography.

Variable pair	Correlation (r)	p-value
Total cholesterol(1)—LDL(1)	0.751	<0.001
Total cholesterol(1)—LDL(2)	0.679	<0.001
Total cholesterol(1)—Remnant cholesterol(2)	-0.409	0.001
LDL(1)—Remnant cholesterol(2)	-0.476	<0.001
LDL(2)—Remnant cholesterol(2)	-0.603	<0.001
Remnant cholesterol(1)—CRP(2)	-0.311	0.015
CK-MB(1)—Troponin-T(1)	0.533	<0.001
CK-MB(2)—Troponin-T(2)	0.694	<0.001
CRP(1)—WBC(1)	0.387	0.002
CRP(2)—WBC(2)	0.315	0.014

Note: (1): Pre-angiography, (2): Post-angiography. Only statistically significant (p<0.05) and clinically relevant correlations are presented. LDL: low-density lipoprotein; CRP: C-reactive protein; CK-MB: creatine kinase-MB; WBC: white blood cell.

The correlations between all biomarkers have been analyzed, and in Table 2, only those correlations that are statistically significant (p<0.05) and considered clinically relevant are presented. According to the table, there was a strong positive correlation between pre-angiography total cholesterol and pre- and post-angiography LDL (p<0.001), supporting the integrity of lipoprotein metabolism. Pre- and post-angiography LDL was inversely correlated with post-angiography remnant cholesterol (p<0.001), suggesting that remnant cholesterol levels may vary independently of LDL. The negative correlation between remnant cholesterol pre-angiography and CRP post-angiography (p=0.015) suggests that remnant cholesterol levels may be related to inflammatory processes.

The strong positive correlation between CK-MB and troponin-T (p<0.001) suggests that markers of myocardial damage act together. The positive correlation between CRP and WBC (p<0.05) supports the effect of the inflammation process on biochemical markers. These findings suggest that remnant cholesterol is associated not only with lipid metabolism but also with inflammatory processes and may be an important marker in the assessment of cardiovascular events.

Regression analysis was performed to evaluate the effect of CK-MB, CRP, troponin-T, and WBC on remnant levels, and the findings are presented in Table 3.

**Table 3:** Multivariable regression analysis of factors affecting remnant cholesterol levels (coefficients expressed per 1 mg/dL change).

Model	Variable	Unstandardized coefficients (β)	Standardized coefficients (β)	T	p-value
Remnant cholesterol(1)	Constant	41.654	–	3.798	**<0.001**
	Troponin-T(2)	-0.014	-0.403	-2.224	**0.031**
Remnant cholesterol(2)	Constant	48.458	–	2.974	**0.004**
	CRP(1)	-1.590	-0.336	-1.740	0.088

Note: (1): Pre-angiography, (2): Post-angiography, troponin-T units: per 1 ng/L, CRP units: per 1 mg/dL. Model 1: Dependent variable=Pre-angiography remnant cholesterol; independent variables=post-angiography, troponin-T, CRP, CK-MB, WBC. Model 2: Dependent variable=post-angiography remnant cholesterol; independent variables=pre-angiography CRP, troponin-T, CK-MB, WBC. CRP: C-reactive protein. Bold values indicate statistical significance at p<0.05.

Regression analyses were conducted to assess statistical associations between variables measured at different time points. The models were not intended to imply causality but to explore potential links between baseline lipid values and subsequent biomarker responses. As indicated in the table, regression analysis revealed that a one-unit increase in post-angiography troponin-T (troponin-T2) significantly decreased pre-angiography remnant cholesterol (remnant1) (β=-0.014, p<0.05). However, no significant effect of troponin-T2 on post-angiography remnant cholesterol (remnant2) was observed (p>0.05). However, this model has been used to describe the relationship between time points rather than establishing a causal relationship. Other variables in the model did not show statistically significant effects on remnant cholesterol. These observational associations may reflect the influence of pre-existing lipid profiles on procedural myocardial susceptibility, though causal inferences are precluded by the study design.

## DISCUSSION

### Remnant cholesterol level and cardiovascular risk

Remnant cholesterol, an independent risk factor for coronary heart disease, significantly increases the risk of myocardial infarction, ischemic stroke, and peripheral artery disease, with a fivefold higher risk for peripheral artery disease^11^. It serves as a key biomarker for long-term risk assessment in acute coronary syndrome and stable coronary artery disease patients, particularly in those with type 2 diabetes, where higher levels correlate with more frequent cardiovascular events^12,13^. Unlike previous studies, our research uniquely evaluates remnant cholesterol levels directly before and after angiography.

Reference values for remnant cholesterol vary, with levels ≥30 mg/dL considered high in acute coronary syndrome and <20 mg/dL optimal for healthy individuals^12,14-16^. Our study revealed a significant rise in remnant cholesterol levels post-angiography, likely due to changes in lipoprotein metabolism and inflammation. These findings emphasize the importance of remnant cholesterol in cardiovascular risk assessment and highlight the need for its monitoring and management to reduce complications.

### Remnant cholesterol and inflammation

Remnant cholesterol increases atherogenic risk by triggering vascular inflammation. Unlike LDL, it accumulates in arterial walls, exacerbating local inflammation and contributing to atherosclerotic cardiovascular disease^17^. Recent studies suggest that combining therapies targeting remnant cholesterol and inflammation may reduce cardiovascular risk^18^. The Copenhagen General Population Study found that each 1 mmol/L (39 mg/dL) increase in remnant cholesterol raises inflammation by 28% and ischemic heart disease risk by 3.3-fold, independent of LDL^7^. In the present study, a negative correlation between post-angiography CRP (CRP2) and pre-angiography remnant cholesterol (remnant1) was observed, though CRP's effect on remnant cholesterol was not statistically significant (p>0.05).

CRP levels significantly increased post-angiography (p<0.05), reflecting a procedure-related inflammatory response. Elevated inflammatory markers post-angiography are linked to adverse clinical outcomes^19,20^, emphasizing the need for regular monitoring of inflammatory biomarkers to mitigate complications. While inflammatory markers showed significant changes, cardiac injury biomarkers like CK-MB demonstrated a different pattern.

### Creatine kinase-MB

In the present study, a significant decrease in CK-MB levels was observed post-angiography (p<0.05). This reduction may reflect decreased cardiac stress, suggesting effective management of myocardial damage post-procedure. Sun et al. demonstrated that reduced cardiac stress correlates with lower CK-MB levels, indicating minimal post-procedural myocardial injury and successful management^21^.

### Other biomarkers

In the present study, HDL levels increased and LDL levels decreased post-angiography, though these changes were not statistically significant (p>0.05). The numerical increase in HDL (p>0.05) may be related to inflammatory processes, but this interpretation should be treated with caution as it falls within the limits of statistical significance. The decline in LDL could indicate acute metabolic adaptations^22^. These trends align with post-angiography metabolic changes, underscoring the importance of lipid regulation in supporting cardiovascular health.

### Correlation analysis

A negative correlation was observed between post-angiography CRP (CRP2) and pre-angiography remnant cholesterol (remnant1), though CRP's effect was not significant (p>0.05). This suggests a non-linear or complex relationship between remnant cholesterol and inflammation, despite literature linking remnant cholesterol to low-grade inflammation^7^. This finding can be explained by two possible clinical scenarios. The first is that chronically high remnant levels may be associated with a diminished inflammatory response in patients with stable atherosclerosis. The second is that low basal remnant levels may reflect transient lipid decreases associated with the acute-phase response due to vascular injury. These interpretations are consistent with the temporal arrangement of measurements (baseline lipids vs. post-procedure CRP) and the exclusion of patients with acute coronary syndrome from the study. Lower baseline remnant cholesterol (remnant1) levels were associated with higher post-angiography troponin T (troponin T2) levels (p<0.05) in our cohort of stable angina patients. This association may be explained by two mechanisms: First, through acute-phase responses, where myocardial injury-induced inflammation temporarily alters lipid metabolism. Second, via plaque vulnerability mechanisms, where lower remnant levels associate with more unstable plaques. However, the pre-procedure timing of remnant measurements precludes causal interpretation. Notably, post-angiography remnant cholesterol (remnant2) showed no correlation with troponin T2, suggesting baseline-specific associations. These findings highlight remnant cholesterol's complex role in lipid metabolism, inflammation, and procedural myocardial susceptibility, warranting future studies on: (1) temporal lipid changes post-angiography and (2) plaque characterization across remnant cholesterol levels.

### Regression analysis

Regression analysis demonstrated an inverse association between post-procedure troponin T (troponin T2) levels and pre-angiography remnant cholesterol (remnant1) (β=-0.014, p<0.05). This temporal relationship suggests that baseline lipid profiles may modulate procedural myocardial susceptibility, consistent with established mechanisms linking remnant lipoproteins to inflammation and cardiac recovery^17,18^. However, other variables did not significantly affect post-angiography remnant cholesterol (remnant2) (p>0.05), and low R-squared values suggest additional factors influence remnant cholesterol levels.

The aforementioned and observed biochemical changes may reflect a combination of procedural effects, underlying patient characteristics, and unmeasured variables. While our findings highlight acute changes in remnant cholesterol post-angiography, uncontrolled confounders and the observational design necessitate cautious interpretation. These regression models describe temporal associations and are not indicative of causal relationships, considering the retrospective nature of the study.

## CONCLUSION

The significant rise in remnant cholesterol post-angiography suggests acute changes in lipoprotein metabolism and inflammation. While LDL levels showed a non-significant decrease, remnant cholesterol's distinct behavior warrants attention. CRP elevation post-procedure did not directly correlate with remnant cholesterol, suggesting complex interactions. The inverse correlation between pre-angiography remnant cholesterol and post-procedure troponin-T (without post-procedure association) implies temporally specific mechanisms. CK-MB reduction may reflect procedural factors. These findings encourage further investigation of remnant cholesterol's pathophysiological role, though clinical utility requires validation in long-term studies.

### Study limitations

This study is limited by its retrospective design and the absence of a control group, which restricts causal inferences. Our interpretations regarding the remnant cholesterol–troponin relationship are constrained by the observational nature of the study and the temporal sequence of measurements. Although changes in biomarkers appear to be associated with the angiographic procedure, it remains unclear whether these changes are a direct result of the intervention or reflect concurrent treatments (e.g., statins, anticoagulants) or the natural course of the disease.

While some parameters (e.g., remnant cholesterol) were elevated post-procedure, we could not determine whether these changes were caused by the angiography itself or by other factors. One limitation of the study is incomplete data for some patients. Demographic and clinical data, including comorbidities, were obtained from electronic health records. However, detailed retrospective records on pre-procedural medications—such as statin doses or adherence to anti-inflammatory treatments—were not accessible.

Another significant limitation is the lack of data on key clinical confounders. Critical information such as medication details (statin dosage, beta-blocker use), renal function parameters (eGFR, creatinine), and anatomical severity of coronary artery disease (number of affected vessels, left ventricular function) was unavailable. These variables could have direct effects on both lipid metabolism and inflammatory responses. Unfortunately, due to the inability to adjust for these factors, we cannot rule out the possibility of residual confounding.

In addition, unmeasured confounders—such as dietary changes or pre-procedural complications—may have influenced the findings. In patients with prior myocardial injury, the effects observed after the procedure may overlap with natural recovery, complicating causal attribution to angiography alone. However, the fact that all patients were evaluated under elective conditions for stable coronary artery disease likely mitigates this concern.

Another limitation is that post-procedural biomarker measurements were obtained at a single time point (18–24 h), preventing a full characterization of their kinetic profiles. Nevertheless, the selected time frame aligns with literature identifying it as optimal for CRP response. Furthermore, the standardized fasting state allows changes in remnant cholesterol to be interpreted independently of nutritional status.

Our findings are hypothesis-generating and warrant confirmation in future prospective, controlled studies. Specifically, studies comparing angiography-exposed and non-exposed patient groups with similar profiles would be valuable to strengthen causal interpretation.

Some biomarkers (e.g., CK-MB, Troponin T) exhibited skewed distributions, especially in the pre-procedural phase. High standard deviations suggest the presence of outliers, likely due to patients with severe myocardial damage. Although non-parametric tests were used, we acknowledge the potential influence of these outliers on the analyses.

A final notable limitation is the absence of long-term clinical outcomes, as our study focused on short-term biomarker changes. It remains uncertain whether the observed post-angiography increases in remnant cholesterol and CRP are transient or persistent and what their implications are in relation to cardiovascular events and risk stratification. Studies exploring the prognostic value of post-procedural remnant cholesterol changes in long-term follow-up are needed.

## ETHICAL APPROVAL

This study was approved by the Ethics Committee of Harran University (18.11.2024, HRÜ/24.18.21) and complied with the Declaration of Helsinki.

## Data Availability

The datasets generated and/or analyzed during the current study are available from the corresponding author upon reasonable request.
